# Differential gene-expression profiles associated with gastric adenoma

**DOI:** 10.1038/sj.bjc.6601399

**Published:** 2004-01-06

**Authors:** H Takenawa, M Kurosaki, N Enomoto, Y Miyasaka, N Kanazawa, N Sakamoto, T Ikeda, N Izumi, C Sato, M Watanabe

**Affiliations:** 1 Department of Gastroenterology and Hepatology, Tokyo Medical and Dental University, Tokyo, Japan; 2 Department of Gastroenterology and Hepatology, Musashino Red Cross Hospital, Tokyo, Japan; 3 Department of Internal Medicine, Yokosuka Kyousai Hospital, Kanagawa, Japan; 4 Department of Health Science, Tokyo Medical and Dental University, Tokyo, Japan

**Keywords:** suppressive subtractive hybridisation, gastric adenoma, gastric adenocarcinoma, expression profile, clustering analysis

## Abstract

Gastric adenomas may eventually progress to adenocarcinomas at varying rates. The purpose of the present study was to identify gene-expression profiles linked to the heterogeneous nature of gastric adenoma as compared to adenocarcinoma. Suppression subtractive hybridisation analysis was performed to extract relevant genes from two cases of low- and high-grade gastric adenomas. The identified genes were quantified by RT–PCR in 14 low-grade adenoma, nine high-grade adenoma and nine adenocarcinoma samples, followed by hierarchical clustering analysis to separate tumours into groups according to their gene-expression profiles. Nine genes previously implicated in carcinogenesis in a variety of organs, including three genes related to gastric adenocarcinoma, were identified. The overexpression of these genes in gastric adenoma has not been reported previously. The clustering analysis of these nine genes across 32 cases identified three groups, one of which consisted primarily of adenocarcinomas, whereas the other two groups consisted of adenomas. One group of adenomas, characterised by larger tumour size, exhibited gene-expression profiles of an intestinal cell lineage implicated in the pathogenesis of an intestinal-type gastric adenocarcinoma. Another adenoma group consisting of low-grade adenomas with smaller tumour size exhibited a unique expression profile. In conclusion, clustering analysis of expression profiles using a limited number of genes may serve as molecular markers for gastric adenoma with different biological properties. Although the prognostic values of these gene-expression profiles need to be evaluated in further follow-up study of adenoma cases, these findings add new insights to (a) our understanding of the pathogenesis of gastric tumours, (b) the development of specific tumour markers for clinical practice, and (c) the design of novel therapeutic targets.

Gastric adenomas are considered to be precancerous lesions, but are clinically heterogeneous, since some may progress to adenocarcinoma, whereas others persist unchanged for long periods (Kamiya *et al*, 1982; Kolodziejczyk *et al*, 1994; Orlowska *et al*, 1995). Identification of adenoma cases with a progressive nature is important since intervention (e.g. endoscopic mucosal resection) is mandatory. Tumour size is a prognostic indicator, but exceptional cases are frequently observed. Histological grading of adenomas as per the Vienna classification (Schlemper *et al*, 2000) has been used to assess the potential for progression. However, exceptional cases are frequent, since 80% of high-grade adenomas progress to adenocarcinomas, whereas 15% of low-grade adenomas progress to high-grade adenomas or adenocarcinomas (Lauwers and Riddell, 1999). Histological diagnosis of biopsy specimens cannot definitively identify adenomas with aggressive potential because sampling errors may contribute to the underestimation of tumour grade or depth of invasion. Thus, an additional prognostic indicator that is independent of conventional clinicopathological findings (e.g. molecular markers) is essential.

Recent comprehensive analyses of gene expression, such as a microarray analysis, identified relevant genes whose expression profiles appeared to be linked to tumour stage, histological grade, susceptibility to chemotherapy, clinical aggressiveness or prognosis (Golub *et al*, 1999; Alizadeh *et al*, 2000; Perou *et al*, 2000; Dhanasekaran *et al*, 2001; Sorlie *et al*, 2001; Shipp *et al*, 2002; van’t Veer *et al*, 2002). Studies on gastric adenocarcinomas revealed several gene-expression profiles that are linked to lymph node metastasis (Hasegawa *et al*, 2002; Hippo *et al*, 2002). Using a similar approach, it may be possible to develop an improved classification scheme for gastric tumours that is capable of distinguishing subgroups of adenomas with progressive natures. Such expression profiles have not been applied to gastric adenomas. In the present study, suppression subtractive hybridization (SSH) analysis (Diatchenko *et al*, 1996; von Stein *et al*, 1997) was used to identify genes relevant to gastric adenomas. Their expression profiles were subsequently assessed in order to identify different progressive potentials of gastric adenomas in comparison to adenocarcinoma.

## MATERIALS AND METHODS

### Tissue sample

Tissue samples of low-grade (6 mm in diameter) and high-grade (15 mm in diameter) gastric adenomas and adjacent gastric mucosa were obtained from 68- and 44-year-old male subjects, respectively, by endoscopic mucosal resection for use in SSH analysis. Additional paired tissue samples of gastric tumours and adjacent mucosa were obtained by endoscopic biopsy or mucosal resection from 14 low-grade adenomas, nine high-grade adenomas and nine adenocarcinomas for gene-expression profiles (Table 1

**Table 1:** Clinical backgrounds of patients

	**Adenoma**		
	**Low grade (*n*=14)**	**High grade (*n*=9)**	**Adenocarcinoma (*n*=9)^a^**
Age (years) (mean±s.d.)	73.5±7.6	67.2±10.8	76.1±9.2
Sex (male/female)	13/1	7/2	7/2
Tumour size (mean±s.d.)^b^	10.1±4.0	19.8±8.3	30.8±12.8

a Adenocarcinoma cases included six cases of T1, two cases of T2 and one case of T3 in TNM clinical classification. Histopathological grading was G1 in seven cases and G2 in two cases.

b *P*<0.005 for low- *vs* high-grade adenoma, low-grade adenoma *vs* adenocarcinoma and *P*<0.05 for high-grade adenoma *vs* adenocarcinoma.

). The nine adenocarcinomas consisted of six T1, two T2 and one T3 tumours based on TNM clinical classification. Histopathological grading was G1 in seven cases and G2 in two cases. Tumour size was significantly different among the low- and high-grade adenomas and the adenocarcinomas. Informed consent was obtained from each patient before biopsy or mucosal resection. The study conformed to the ethical guidelines of the 1975 Declaration of Helsinki and was approved by the IRB.

### RNA extraction and SMART™ cDNA synthesis

Total RNA was extracted by the modified acid–guanidium–chloroform method (Chomczynski and Sacchi, 1987) using ISOGEN™ (Nippon Gene, Toyama, Japan). Full-length cDNAs were generated from the total RNA using the SMART™ (Switch Mechanism at 5′ end of RNA Template) PCR cDNA synthesis kit (Clontech, Palo Alto, CA, USA) (Matz *et al*, 1999) following the manufacturer’s instruction and used for SSH analysis. Quantitative analysis of specific genes was performed on cDNAs generated from 1 *μ*g of total RNA in 10 *μ*l mixture with 200 U of Superscript™ reverse transcriptase (Gibco, Madison, WI, USA) using random hexamer primers.

### Suppression SSH and sequencing

Subtractive hybridization was performed using a PCR-Select™ cDNA subtraction kit (CLONTECH, Tokyo, Japan) according to the manufacturer’s instructions. Briefly, after two different adaptors were ligated to *Rsa*I-digested SMART™ cDNA from the gastric adenoma tissues (tester), 2.5 ng of each adaptor-ligated SMART™ cDNA was hybridised with 1.5 *μ*g of *Rsa*I-digested SMART™ cDNA from the adjacent gastric mucosa (driver). In this process, cDNA sequences specific to the tester were enriched. A total of 10 ng of PCR products were cloned into plasmids pGEM-T Easy Vector™ (Stratagene, Cedar Creek, TX, USA) and transformed to competent *Escherichia coli* XL2-blue™ Ultracompetent cells (Gibco, Madison, WI, USA). In all, 100 colonies were randomly picked and sequenced using the PRISM dye termination kit™ (ABI, Chiba, Japan). BLAST Search 2.0 (www.ncbi.nlm.nih.gov/blast/bla st.cgi) was used to analyse sequence homologies in the gene database.

### Quantitative analysis of identified genes

Overexpression of genes identified by SSH was verified in the original samples by semiquantitative RT–PCR using gene-specific primer sets. The PCR products were obtained during the exponential phase of amplification and the amounts of products were compared by agarose-gel electrophoresis. Subsequently, the mRNA expression levels of these genes were quantitated using real-time PCR (Light Cycler System™, Roche Diagnostics, Manheim, Germany) (Wittwer *et al*, 1997). The expression level of the target gene was standardised with that of the house-keeping beta-actin gene and the ratio of each gene expression in paired samples (adenoma or adenocarcinoma/adjacent mucosa) was calculated. The primers used in the quantitative PCR were as follows: acyl-CoA binding protein (*ACBP*)-sense, 5′AgTTTgAgAAAgCTgCAgAggAgg3′; *ACBP*-antisense, 5′TCCCgAATTCCCACCATCCACggT3′; eukaryotic elongation factor 1 gamma (*EEF1G)*-sense, 5′TATCgCTTCCCTgAAgAACTCACT3′; *EEF1G*-antisense, 5′TCgCTgCCAggATCCAgTTTCCgC3′; peripheral-type benzodiazepine receptor (*BZRP*)-sense, 5′gCgACCACACTCAACTACTgCgTA3′; *BZRP*-antisense, 5′gCATgCAGAAAgCACAggACACTg3′; arginase II (*ARG2)*-sense, 5′gAgACAAAgACCAATCCATTTgA3′; *ARG2*-antisense, 5′gTgTATTTCCTCAgCAATATACAT3′; histone H2A.Z (*H2AFZ)*-sense, 5′TggCAggAAATgCATCAAAAgACT3′; *H2AFZ*-antisense, 5′ggAAAgCTAATTAAACTTCCAACT3′; *GW112*-sense, 5′gAATCTTCTACCTCATAACTTCCT3′; *GW112*-antisense, 5′gCAACAACTgATACACTCATAAgT3′; pepsinogen C (*PGC*)-sense, 5′CAGCTTGACCTTCATCATCAATG3′; *PGC*-antisense, 5′CCAGAGTGGAAAGACAGATACAA3′; defensin alpha 5 (*DEFA5)*-sense, 5′ATCCTTgCTgCCATTCTCCTggTg3′; *DEFA5*-antisense, 5′ACCTgAggTTCTAAgAgCAgAgA3′; receptor for activated C-kinase (*RACK1)*-sense, 5′AACAgCAAgCAACCCTATCATCgT3′; *RACK1*-antisense, 5′gATAACTTCTTgCTTCAgTTCATC3′; LI-Cadherin (*CDH17*)-sense, 5′AACTTAACgATAgAggTgTCTgAC3′; and *CDH17*-antisense 5′gCTTTgAACACAATgTTggAAACA3′.

When the expression levels of target genes were below the sensitivity of the assay, the detection limits for each gene were substituted to calculate the ratio. The ratio was not determined when the target gene could not be quantitated in both the paired samples. For example, the expression level of *GW112* was below the sensitivity of this quantitation system in all samples and, therefore, this gene was not included in subsequent analyses.

### Analysis of gene-expression profiles

Unsupervised hierarchical clustering analysis was performed based on similarities of gene expression using web-available software (Expression Profiler; European Bioinformatics Institute; http://ep.ebi. ac.uk/). The ratio of each gene expression in paired samples was log transformed and applied to the clustering algorithm.

### Statistical analyses

Data were compared using the *χ*^2^ test or Fisher’s exact test. Distributions of continuous data were analysed by the Mann–Whitney *U-*test or Student’s *t*-test between two groups and by ANOVA with adjustment for multiple comparison by Scheffe’s method among three groups using Statview 5.0 software (Abacus Concepts, Berkeley, CA, USA).

## RESULTS

### SMART™ RT–PCR and SSH

**Figure 1:**
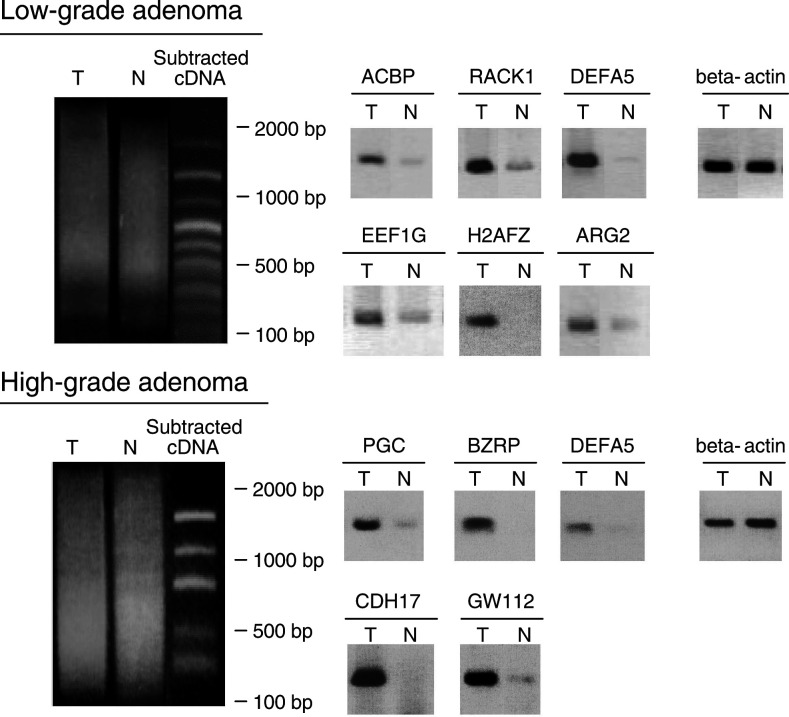
Electrophoretic band patterns of SSH and gene overexpression in original tester tissue. (**A**) The cDNA amplified by SMART™ RT–PCR and cDNA after subtraction by SSH were electrophoresed on 2.0% agarose. The amplified cDNA derived from gastric adenoma and adjacent gastric mucosa appears as a smear before SSH. After SSH, it exhibits several distinct bands. (**B**) Semiquantitative RT–PCR using gene-specific primer sets for each identified gene were performed. PCR products were analysed at the PCR cycle number in the exponential phase of amplification (*ACBP* 36 cycles, *RACK1* 27 cycles, *DEFA5* 36 cycles, *EEF1G* 13 cycles, *H2FAZ* 30 cycles, *ARG2* 36 cycles, *PGC* 25 cycles, *BZRP* 24 cycles, *CDH17* 22 cycles, *GW112* 40 cycles, *beta-actin* 20 cycles). The expression level of housekeeping gene (*beta-actin*) was at the same level, but those of genes identified by SSH were clearly more abundant in low- and high-grade gastric adenoma tissues compared to their corresponding adjacent mucosa. T and N indicate gastric tumour and adjacent normal mucosa, respectively.

The cDNA generated by SMART™ RT–PCR exhibited a smear pattern representing amplification of the whole mRNA species on agarose-gel electrophoresis, whereas after SSH it consisted of several discrete bands derived from differentially expressed genes (Figure 1

). Nucleotide sequencing was performed for 100 independent clones for each SSH experiment. Six genes represented more than once in low-grade adenoma samples: (a) *RACK1* (accession number NM_001172), (b) *ARG2* (NM_001172), (c) *EEF1G* (NM_001404), (d) *ACBP* (NM_02054), (e) *H2AFZ* (NM_00210) and (f) *DEFA5* (NM_021010). In addition, five genes represented more than once in high-grade adenoma samples: (a) PGC (NM_002630), (b) *BZRP* (NM_007311), (c) *DEFA5*, (d) *GW112* (AF097021), and (e) *CDH17* (NM_004063) (Table 2

**Table 2:** Genes identified by SSH analysis

**Gene**	**Symbol**	**Accession no.**
*Low-grade adenoma derived*		
Receptor for activated C-kinase	RACK1	NM-006098
Arginase II	ARG2	NM-001172
Eukaryotic elongation factor 1 gamma	EEF1G	NM-001404
Acyl-CoA binding protein	ACBP	NM-020548
Histone H2A.Z	H2AFZ	NM-002106
Defensin alpha 5	DEFA5	NM-021010
		
*High-grade adenoma derived*		
Pepsinogen C	PGC	NM-002630
Peripheral-type benzodiazepine receptor	BZRP	NM-007311
Defensin alpha 5	DEFA5	NM-021010
GW112	GW112	AF097021
LI-Cadherin	CDH17	NM-004063

). *Defensin alpha 5* was detected in both the SSH samples. All these genes have been previously implicated in carcinogenesis in different organs or cell proliferation. These repetitively detected genes were used for further analysis and the miscellaneous genes that were detected only once were excluded.

### Confirmation of differential gene expression by semiquantitative RT–PCR

The overexpression of genes in SMART-cDNA samples was verified by semiquantitative RT–PCR using gene-specific primer sets. PCR products isolated during the exponential phase of amplification were analysed by agarose-gel electrophoresis in order to compare the amount of specific products. The minimal number of PCR cycles required for visualisation on agarose gels was selected for each gene. The amount of PCR product at the same PCR cycle was similar for beta-actin, a representative housekeeping gene, but those of genes identified by SSH were clearly more abundant in the gastric adenoma or adenocarcinoma tissues (Figure 1 in comparison to their corresponding adjacent gastric mucosa tissues.

### Quantification of identified genes

**Figure 2:**
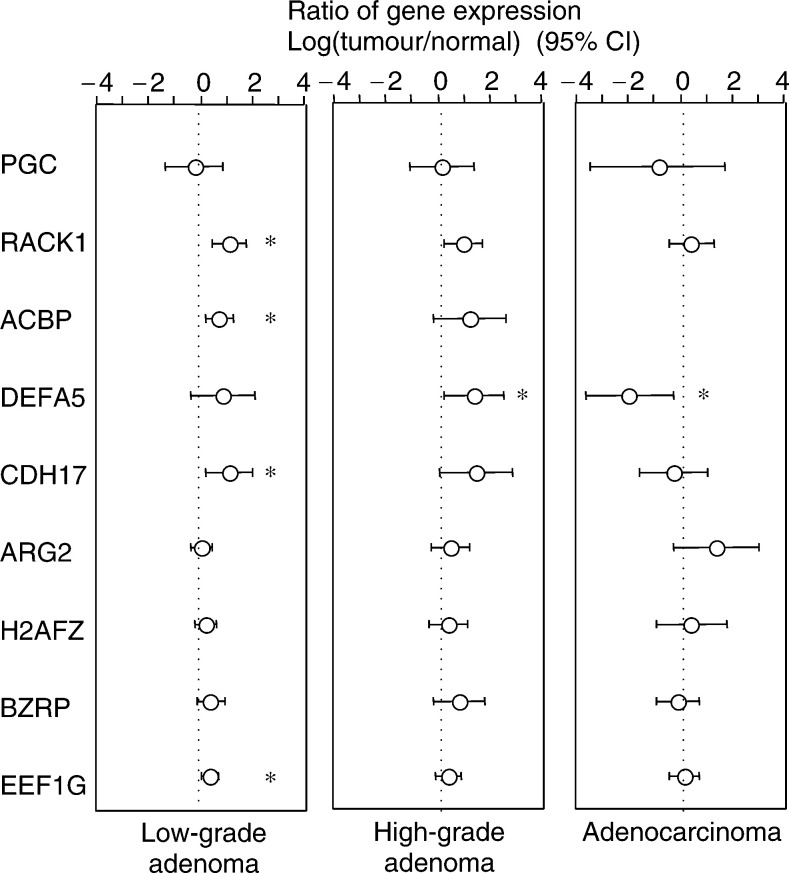
Expression levels of identified genes in 32 cases. The expression levels of nine genes in 14 low-grade adenomas, nine high-grade adenomas and nine adenocarcinomas are shown as the mean and 95% confidence interval. Data for *ACBP* in adenocarcinomas were not available for analysis due to the small number of cases. *RACK1, ACBP, CDH17* and *EEF1G* were significantly overexpressed in low-grade adenoma; *DEFA5* was significantly overexpressed in high-grade adenoma and suppressed in adenocarcinoma.

The expression levels of the identified genes were quantified by quantitative PCR in 14 low-grade adenomas, nine high-grade adenomas and nine adenocarcinomas, including the original samples used in SSH analyses (Figure 2

). *RACK1, ACBP, CDH17* and *EEF1G* were significantly overexpressed in low-grade adenomas, whereas *DEFA5* was significantly overexpressed in high-grade adenomas and suppressed in adenocarcinomas. These findings suggest that these genes reflect the molecular features of gastric tumours with different histological diagnoses, but that individual analysis of these genes does not define the progressive potential of gastric tumours.

### Analysis of gene-expression profiles using unsupervised hierarchical clustering

**Figure 3:**
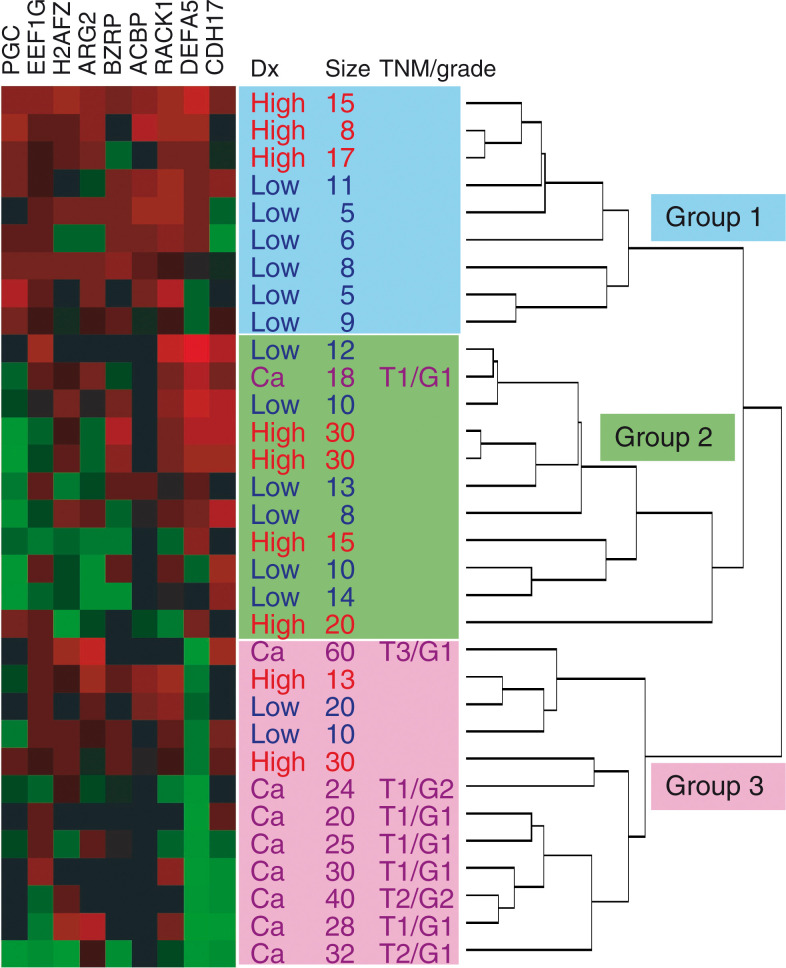
Hierarchical clustering analysis. The expression patterns of nine genes across 32 samples are shown. Each column indicates a gene, and each row indicates a sample. Red and green indicate the overexpression and underexpression, respectively, of genes in adenomas or adenocarcinomas in comparison to the adjacent mucosa. Graduated colour patterns correspond to the degrees of expression changes. Black colour indicates that the expression was not detected in both the paired samples. The dendrogram of the 32 cases at the right of the matrix, in which the pattern and length of branches reflect the relatedness of the samples, indicates that the samples are clustered into three major branches based on the similarity of gene-expression profiles. The abbreviations Low, High and Ca stand for low-grade adenoma, high-grade adenoma and adenocarcinoma, respectively.

In order to determine if the analysed samples could be classified into groups on the basis of their gene-expression profiles alone, hierarchical clustering analysis was performed. The ratio of gene expression was first log transformed and then applied to the clustering algorithm. The expression patterns of nine genes across 32 samples are shown in Figure 3

. The dendrogram of the 32 cases at the right of the matrix, in which the pattern and length of branches reflect the relatedness of the samples, separated samples into three major groups based on the similarities in gene-expression profiles.

### Clinicopathological factors in relation to clustered groups

In order to clarify the clinical features associated with this clustering, various clinicopathological factors, including age, gender, histological diagnosis and tumour size, were analysed. The proportions of low- and high-grade adenomas or adenocarcinomas were significantly different among the three groups (*P*<0.05) (Table 3

**Table 3:** Clinical backgrounds of all cases categorised into three groups by cluster analysis

	**Group-1 (*n*=9)**	**Group-2 (*n*=11)**	**Group-3 (*n*=12)**
Age (years) (mean±s.d.)	69.4±10.7	68.9±7.1	78.0±8.1
Sex (male/female)	9/0	9/2	9/3
Histological diagnosis^a^	6/3/0	6/4/1	2/2/8

*Tumor size (mean±s.d.)*^b^	9.3±4.3	16.4±7.6	27.7±13.1
Adenoma^c^	9.3±4.3	16.2±8.0	18.3±8.9
Adenocarcinoma *(adenocarcinoma cases only)*		18.0	32.4±12.7
TNM clinical classification (T1/T2 or T3)		1/0	5/3
Histological differentiation (G1/G2)		1/0	6/2

a Low-grade adenoma/high-grade adenoma/adenocarcinoma according to Vienna classification. *P*<0.05.

b *P*<0.001 for group-1 *vs* 3 and *P*<0.05 for group-1 *vs* 2, group-2 *vs* 3.

c *P*<0.05 for group-1 *vs* 2 and for group 1 *vs* groups 2 and 3 collectively (16.8±8.0).

). The first group consisted of six low-grade and three high-grade adenomas; the second group consisted of six low-grade and five high-grade adenomas and an adenocarcinoma; the third group consisted of eight adenocarcinomas, two low-grade adenomas and two high-grade adenomas. Three cases of adenocarcinoma in advanced tumour stage (T2 or T3 in TNM classification) and two cases with moderately differentiated adenocarcinoma (G2) clustered into the third group. When adenomas and adenocarcinomas were analysed together, tumour size became significant in the order of groups 1–3. The tumour size of adenomas was significantly small in group 1 in comparison to group 2 or to groups 2 and 3 collectively. When low- and high-grade adenomas were compared separately, the tumour size of low-grade adenomas in group 1 was significantly smaller in comparison to those in group 2 or in groups 2 and 3 collectively. High-grade adenomas in group 1 were smaller than those in group 2 or in groups 2 and 3 collectively, although the difference was not statistically significant (Figure 4

**Figure 4:**
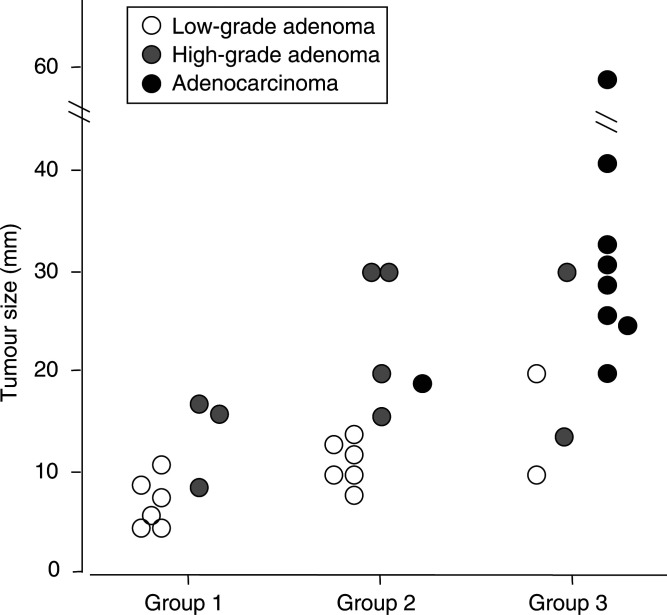
Plot of the tumour size according to groups separated by clustering analysis. Open circles denote low-grade adenomas, grey circles denote high-grade adenomas and closed circles denote adenocarcinomas. Tumour size was significantly larger in group 3 in comparison to groups 1 or 2 and significantly larger in group 2 *vs* group 1. When a comparison was made independent of histological diagnosis, tumour size of low-grade adenomas was significantly smaller in group 1 in comparison to group 2 or to groups 2 and 3 collectively (*P*<0.05). High-grade adenoma in group 1 also tended to be smaller than those in group 2 or groups 2 and 3 in combination, although the differences did not reach statistic significance (*P*=0.09 for group 1 *vs* 2 and *P*=0.10 for group 1 *vs* groups 2 and 3 collectively).

).

### Gene-expression profiles with respect to clustered groups

**Figure 5:**
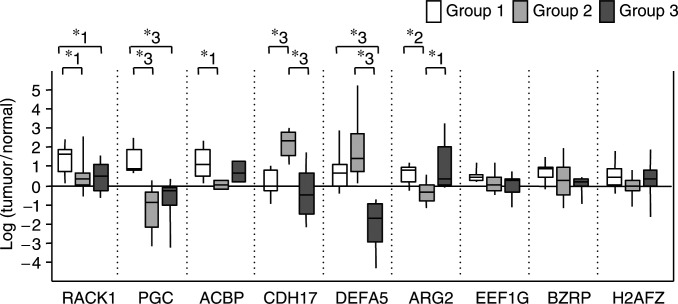
Plot of the gene-expression ratio according to groups separated by clustering analysis. The gene-expression ratios were box plotted according to three groups identified in clustering analysis. The expression levels of *ACBP*, *PGC* and *RACK1* were significantly high in group 1 compared to group 2 and/or group 3. In contrast, the expression levels of *CDH17* and *DEFA5* were significantly high in group 2 compared to group 1 or group 3. Only *ARG2* showed a high level of expression in group 3. ^*^1 *P*<0.05, ^*^2 *P*<0.01, and ^*^3 *P*<0.005.

In order to investigate the gene-expression profiles responsible for this clustering, the expression levels of each gene were compared between each of the three groups by the Mann–Whitney *U*-test. The expression levels of *ACBP*, *PGC* and *RACK1* were significantly higher in group 1 in comparison to group 2 and/or group 3 (*ACBP*: *P*<0.05 for 1 *vs* 2; *PGC: P*<0.001 for 1 *vs* 2, *P*<0.005 for 1 *vs* 3; *RACK1*: *P*<0.05 for 1 *vs* 2 and 1 *vs* 3). In contrast, the expression levels of *CDH17* and *DEFA5* were significantly higher in group 2 in comparison to groups 1 or 3 (*CDH17*: *P*<0.005 1 *vs* 2 and 2 *vs* 3; and *DEFA5*: *P*<0.0001 for 2 *vs* 3 and *P*<0.0005 for 1 *vs* 3). Only *ARG2* exhibited a high level of expression in group 3 (*P*<0.05 for 2 *vs* 3, *P*<0.01 for 1 *vs* 2). The hierarchical clustering analysis using these six genes resulted in clusters identical to that using nine genes (data not shown). The plot of the log-transformed ratio of these genes is shown in Figure 5

.

## DISCUSSION

In the present study, we identified nine genes specifically overexpressed in low- and high-grade gastric adenomas. Although these genes have been implicated in carcinogenesis in a variety of organs, the overexpression of these genes in gastric adenomas has not been investigated previously. Unsupervised clustering analysis of expression profiles using these gastric adenoma-related genes was performed in a total of 32 gastric adenomas and adenocarcinomas, resulting in a classification with a close correlation to histological stages. Moreover, the adenomas were further divided into two subgroups with different tumour sizes according to their expression profiles. These results suggest that expression profiles may be linked to different biological properties of gastric adenomas or adenocarcinomas.

Analysis of the nine adenoma-related genes in 32 cases of gastric tumours demonstrated that a portion of the genes exhibited significantly increased expressions in adenomas, whereas none of these genes was overexpressed in adenocarcinomas (Figure 2). This suggests that these genes play a specific role in the development of adenomas, but that their expression levels were variable in these tumours. This observation raises the possibility that the molecular nature of gastric adenomas is heterogeneous and separate analyses of individual genes are not informative. Accordingly, we tried to classify gastric tumours using gene-expression profiling.

Three distinct groups of gastric tumours were identified by an unsupervised hierarchical clustering analysis of expression profiles of nine adenoma-related genes. A search for clinicopathological features linked to this classification revealed that these three groups differed significantly in their constitutive proportions of low- and high-grade adenomas or adenocarcinomas. One group consisted predominantly of adenocarcinomas (group 3) into which all advanced clinical stage or histological grade adenocarcinomas were classified, suggesting that expression profiles successfully distinguished gastric adenocarcinomas from adenomas.

The other two groups (groups 1 and 2) consisted of mixtures of low- and high-grade adenomas. Group 1 adenomas were significantly smaller than group 2 tumours, demonstrating that the expression profiles differentiate gastric adenomas into two subgroups with potentially different biological properties undetected by conventional histopathological classification. We suggest that gene-expression profiles not only confirm major histologic distinctions between gastric adenomas and adenocarcinomas but may also define subgroups of gastric adenomas with different biological natures.

Group 1, consisting of small adenomas with a slightly increased proportion of low-grade cases and no adenocarcinomas, exhibited expression profiles characterised by three overexpressed genes, that is, *RACK1*, *PGC* and *ACBP*. *RACK1*, previously known as G protein beta-subunit-like protein 12.3, is a signal molecule involved in the MAPK pathway through binding to Src, integrin beta-subunit or interferon receptor (Chang *et al*, 1998; Liliental and Chang, 1998; Croze *et al*, 2000; Kiely *et al*, 2002). *Pepsinogen C* is the precursor of pepsin C that is expressed in the normal gastric mucosa, and is also involved in gastric epithelial cell growth during mucosal healing (Kishi *et al*, 1997). *Acyl-CoA binding protein* is involved in steroid biosynthesis and in the stimulation of cell proliferation (Papadopoulos, 1993). Although these genes are related to cell proliferation and their overexpression has been reported in tumours of different organs, their association with gastric adenocarcinomas has not been confirmed (Diez-Itza *et al*, 1993; Miettinen *et al*, 1995; Vizoso *et al*, 1995; Konishi *et al*, 1999; Venturini *et al*, 1999; Berns *et al*, 2000; Miyasaka *et al*, 2001; Saito *et al*, 2002). In the present study, the expression of these genes did not increase in either group 2, the larger adenomas or group 3, the adenocarcinomas. These genes may play a role in the pathogenesis of gastric adenomas in their early stages or in more benign courses, that is, a limited role in the progression to adenocarcinoma.

In comparison, group 2, a mixture of larger low- and high-grade adenomas, as well as one adenocarcinoma, was characterised by the overexpression of two intestine-specific genes, *CDH17* and *DEFA5*. LI-Cadherin is usually expressed in normal intestinal mucosa and ectopically in well-differentiated gastric adenocarcinomas (Grotzinger *et al*, 2001). *Defensin alpha 5* consists of a family of antimicrobial peptides that are highly expressed in small intestinal Paneth cells (Inada *et al*, 2001). The defensin family has alternative functions, such as promotion of cell differentiation (Frye *et al*, 2001). It is also known to be overexpressed in cancers of the kidney and oral mucosa (Muller *et al*, 2002). The intestine-specific transcription factor, CDX2, has recently been implicated in the regulation of *CDH17* and *DEFA5* (Eda *et al*, 2002; Hinoi *et al*, 2002). In the normal small intestine, *CDX2* controls the expression of genes that determine the cellular lineage of the small intestinal epithelium. The ectopical expression of CDX2 has been reported in intestinal-type gastric adenocarcinomas (Almeida *et al*, 2003), and this is consistent with microarray data suggesting that a group of intestine-specific genes are upregulated in gastric adenocarcinomas (Hippo *et al*, 2002). Collectively, the upregulation of two *CDX2*-dependent genes, *CDH17* and *DEFA5*, found in group 2, represents a characteristic of intestinal cellular lineage that is, in the stomach, implicated in the pathogenesis of intestinal-type gastric adenocarcinoma.

In group 3, which consisted mainly of gastric adenocarcinomas, only *ARG2* exhibited a high level of expression. *Arginase II* has been reported to be overexpressed in cancerous tissues in general (Harris *et al*, 1983; Leu and Wang, 1992; Suer Gokmen *et al*, 1999; del Ara *et al*, 2002; Porembska *et al*, 2003) and it is well established that this gene is overexpressed in gastric adenocarcinomas (Wu *et al*, 1996). Since *ARG2* catalyses the conversion of arginine to ornithine, a crucial metabolite in biosynthesis of glutamic acid, proline and polyamines (Vockley *et al*, 1996), an increase in the level of arginase may reflect accelerated metabolism due to cell proliferation or tumour growth. Therefore, the overexpression of *ARG2* in adenocarcinomas, as defined in the present study, is reasonable.

Genes other than those listed above were sporadically overexpressed in a portion of the adenomas or adenocarcinomas, although their expression levels were not significantly different among the three groups. *Eukaryotic elongation factor 1 gamma* is a subunit of EF1 and it is involved in RNA translation (Janssen *et al*, 1991). *Histone H2A.Z* is a histone protein of the H2A family and it is involved in DNA replication (Hatch and Bonner, 1990). *Peripheral-type benzodiazepine receptor* is involved in mitochondrial cholesterol transport and proliferation, steroid biosynthesis, and the stimulation of cell proliferation (Papadopoulos, 1993). The overexpression of these genes is sporadic in cancers of a variety of tissues (Lew *et al*, 1992; Miettinen *et al*, 1995; Mimori *et al*, 1995, 1996; Mathur *et al*, 1998; Hardwick *et al*, 1999; Venturini *et al*, 1999). Hierarchical clustering analysis excluding *EEF1G*, *H2AFZ* and *BZRP* resulted in clusters identical to that using the original nine genes, suggesting that these three genes do not contribute to the molecular classification of three groups, but may be involved in the common pathophysiology of gastric tumours probably reflecting accelerated cell division or metabolism.

Recent studies using a microarray analysis defined gene-expression profiles of gastric adenocarcinoma (Hasegawa *et al*, 2002; Hippo *et al*, 2002). Interestingly, the spectra of genes that were overexpressed in carcinoma tissues in these studies differ significantly from the present study. The possible reason for this discrepancy may be that these microarray studies analysed advanced staged gastric carcinoma tissues. Since the samples used for the extraction of relevant genes in the present study were low- and high-grade adenomas, the detected genes may be overexpressed specifically in adenoma tissues and not in adenocarcinoma tissues. Thus, it seems reasonable that advanced staged adenocarcinoma possess different gene-expression profiles from those obtained in the present study. To elucidate the stage-specific gene expressions, different stages of gastric tumours should be analysed.

The results of the present study raise the possibility that the expression profiles of specific genes may distinguish gastric adenomas from adenocarcinomas and, more importantly, may define subgroups of gastric adenomas that are unresolved by conventional histopathology. Many studies have shown that gene-expression profiles can be used to identify tumour subclasses independent of histopathological diagnosis. Furthermore, these tumour subclasses are frequently related to distinct cellular lineages and are closely associated with prognosis or response to treatment as shown in malignant lymphomas (Alizadeh *et al*, 2000; Shipp *et al*, 2002) or breast cancer (Sorlie *et al*, 2001), confirming the usefulness of expression profiling in the clinical practice of cancer. Group 2, a mixture of low-and high-grade adenomas cases with larger tumour sizes, exhibited gene-expression profiles specific to the cellular lineage of intestinal epithelium that has been implicated in an intestinal-type gastric adenocarcinoma (Hippo *et al*, 2002). Thus, adenomas classified as group 2 tumours may have a biological nature more closely related to adenocarcinomas in comparison to the adenomas classified in group1. On the one hand, the expression profiles of *CDH17, DEFA5* and other *CDX2* regulated genes may constitute specific tumour markers for a distinct subgroup of gastric adenomas with a progressive nature. On the other , gastric adenomas with expression profiles similar to those of the smaller adenomas in group 1 may be nonprogressive. There are no definite histological or clinical markers to identify the progressive subgroup of adenomas. Therefore, future applications of expression profiling of these genes in biopsied samples may contribute to clinical practice and may promote objective criteria for intervention, such as endoscopic mucosal resection.

However, there are several limitations in the present study including that it is cross-sectional. There is no follow-up of the adenoma cases and no data available on the prognoses or the disease progression of the adenoma cases. Thus, the actual prognostic value of this classification remains to be elucidated. A longitudinal study is necessary to determine if adenomas classified into group 2 actually develop into progressive diseases. These types of studies are particularly difficult because lesions diagnosed histologically as high-grade adenomas are resected endoscopically without follow-up, as recommended in the literature (Lauwers and Riddell, 1999). Another issue is that expression profiling is not in complete accord with conventional histopathological classification, (e.g. three high-grade adenomas classified into group 1 or two low- and high-grade adenomas classified into group 3). Nevertheless, we believe that more accurate discrimination will be achieved by increasing the number of predictive genes involved in expression profiling by extracting them through more comprehensive investigations of gene expression, such as a large-scale DNA microarray analysis. Alternatively, the detailed molecular and pathological analyses of exceptional cases may provide additional predictive information on the biological nature of gastric tumours. All the three high-grade adenomas in group 1 were less than 20 mm in diameter (8, 15 and 17 mm) and an adenocarcinoma in group 2 is the smallest T1/G1 tumour, raising the possibility that such exceptional cases have particular biological characteristics below the sensitivity of conventional histopathological examination. Their discrimination may be achieved by gene-expression profiling.

In conclusion, taking advantage of the expression profiles of a set of genes identified in two cases of gastric adenoma, gastric adenoma and adenocarcinoma can be classified into three groups with distinct gene-expression patterns. One group consists primarily of invasive adenocarcinoma, whereas the other two groups consist of adenomas with potentially different biological properties, as suggested by significantly different tumour sizes. These findings add new insight into our understanding of the molecular pathogenesis involved in the early stages of gastric carcinogenesis, in developing specific tumour markers for clinical practice and in designing potentially novel therapeutic targets.
